# Supplementary material for: The miRNA Profile in Non-Hodgkin’s Lymphoma Patients with Secondary Myelodysplasia
Source: Cells. 2020 Oct 19;9(10):2318. doi: 10.3390/cells9102318 (PMC7656297; doi:10.3390/cells9102318)
Supplement: Supplementary file 1 [file cells-09-02318-s001.pdf]

Table S1. Patient characteristics at the time of diagnosis.

non-Hodgkin's lymphomas (n=41)

		DLBCL				
Factor		ABC	GBC	MCL	CLL/SLL	FL
Gender						
	Male	16	1	3	2	1
	Female	8	4	2	4	
Age (years)						
	>60	14	1	3	4	
	<60	10	4	2	2	1
LDH						
	Increased	17	4	4	4	
	Normal	7	1	1	2	1
B symptoms						
	Yes	24	4	5	4	
	No		1		2	1
Clinical stage						
	I+II	11	2	1		
	III+IV	13	3	4	6	1
IPI score						
	High-risk	24		4	1	
	median high-risk		5		1	1
	Low-risk			1	4	
Myelodysplasia						
	Yes	10	1	4	3	1
	No	14	4	1	3	
Anemia						
	Yes	7	1			
	No	17	4	5	7	

DLBCL, diffuse large B cell lymphoma;
ABC, activated B-cell like; GBC, germinal center B-cell like;
MCL, mantle cell lymphoma;
CLL/SLL, chronic lymphocytic leukemia/small lymphocytic lymphoma;
LDH, lactate dehydrogenase; IPI, International Prognostic Index.

Myelodysplastic syndromes (n=19)

Factor	Value
Gender	
Male	0
Female	10
Age (years)	
>60	15
<60	4
Hemoglobin, g/L	80(72-91)
WBC count, × 10^3/L	4,9(4-6,25)
ANC, /dL	4(2,9-5,3)
Circulating blast count, %	0(0-4)
Platelet count, × 10^3/L	140(97,5-182)
LDH (conversion), mg/L	188(128,25-380)
Bone marrow cellularity, %	70(55-75,25)
Bone marrow blasts, %	<5
MDS subtype	
RCUD	8
RAEB	3
RCMD	3
RARS	4
MDS-U	1
Cytogenetic risk group	
Good	
Normal	9
Intermediate	7
Poor	3
R-IPSS risk category	
Very low	7
Low	3
Intermediat	4
High	2
Very high	3

Values are presented as number (%) or median (range).
WBC, white blood cells; ANC, absolute neutrophil count; LDH -Lactate dehydrogenase;
MDS, myelodysplastic syndrome; RCUD, refractory cytopenia with unilineage dysplasia;
RARS, refractory anemia with ringed sideroblasts; RCMD, refractory cytopenia with multilineage dysplasia;
RAEB, refractory anemia with excess of blasts; MDS-U, myelodysplastic syndrome-unclassifiable;
R-IPSS, revised international prognostic scoring syste

non-cancerous blood diseases (n=58)

Factor	Value
Gender	
Male	23
Female	35
Age (years)	
>60	11
<60	47
Hemoglobin, g/L	90(76,25-110)
WBC count, × 10^3/L	6,7(4,7-9,1)
ANC, /dL	5(3,2-7)
Platelet count, × 10^3/L	200,5(125,75-280)
Iron-deficiency anemia	33
hemolytic anemia	3
B12 deficiency anemia	5
chronic disease anemia	6
immune thrombocytopenia	10
aplastic anemia	1

Values are presented as median (range).

Table S2. The sequences of primers and miRNA probes.

Target	Type	Sequence (5' → 3')
miR-145-5p	RT primer	CGTGTCGCCTTGTAGCACGACCTTATTCGCACCCTCGACACGA CAGGGATTC
	Reverse PCR primer	GCCTTGTAGCACGACCTTA
	PCR probe	(R6G)-TTCGCACCC(T-BHQ1)CGACACGACAGGGATTC
	Forward PCR primer	ACACGTCCAGTTTTCCCAG
miR-155-5p	RT primer	GTCAGAGCGCTCTTCTAGCACCACTCTATCCTACCCTCGCTCT GACACCCCTA
	Reverse PCR primer	GCTCTTCTAGCACCACTCTA
	PCR probe	(R6G)-TCCTACCC(T-BHQ1)CGCTCTGACACCCCTA
	Forward PCR primer	CCCAGCTTAATGCTAATCGTGA
miR-451a	RT primer	GTCGTGTCTGAGGCTCACTGAGACCTATTCGCACCTCGACACG ACAACTCAGT
	Reverse PCR primer	CTGAGGCTCACTGAGACCT
	PCR probe	(R6G)-ATTCGCACC(T-BHQ1)CGACACGACAACTCAGTA
	Forward PCR primer	CCAGCAAACCGTTACCATT
let-7a-5p	RT primer	GTCGTGTCTGAGGCTCACTGACACAATTCGCACCCTCGACACG ACAACTATAC
	Reverse PCR primer	CTGAGGCTCACTGACACAA
	PCR probe	(R6G)-C+C+C+T+CGA+CACGACAACTATAC-(BHQ1)
	Forward PCR primer	CAGCACTGAGGTAGTAGGTT
miR-1246	RT primer	CGTGTCGCCTTGTAGCACGACCTTATTCGCACCTCGACACGAC CTCCTGCT

	Reverse PCR primer	GCCTTGTAGCACGACCTTA
	PCR probe	(R6G)-TTCGCACC(T-BHQ1)CGACACGACCTCCTGCT
	Forward PCR primer	ACACGCGAATGGATTTTTGG
miR-126-3p	RT primer	GTCGTGTCTGAGGCTCACTGAGACCTATTCGCACCTCGACACGACCGCATTATT
	Reverse PCR primer	CTGAGGCTCACTGAGACCT
	PCR probe	(R6G)-ATTCGCACC(T-BHQ1)CGACACGACCGCATTATT
	Forward PCR primer	TCTCACTCGTACCGTGAGT
miR-150-5p	RT primer	GTCGTGTCGTGAAGCAGACAGACACAATCGCACCTCGACACGACCACTGGTAC
	Reverse PCR primer	GTGAAGCAGACAGACACAA
	PCR probe	(R6G)-TCGCACC(T-BHQ1)CGACACGACCACTGGTAC
	Forward PCR primer	CACGCTCCGACTCTCCCAACCCTT
miR-16-5p	RT primer	GTCGTGTCTGAGGCTCACTGAGACCTATTCGCACCTCGACACGACCGCCAATA
	Reverse PCR primer	CTGAGGCTCACTGAGACCT
	PCR probe	(R6G)-ATTCGCACC(T-BHQ1)CGACACGACCGCCAATA
	Forward PCR primer	CCAGCTAGCAGCACGTAAA
miR-181a-5p	RT primer	GTCGTGTCTGAGGCTCACTGAGACCTATTCGCACCTCGACACGACACCCACCG
	Reverse PCR primer	CTGAGGCTCACTGAGACCT
	PCR probe	(R6G)-ATTCGCACC(T-BHQ1)CGACACGACACCCACCG
	Forward PCR primer	CCAGCAACATTCATTGCTGT
miR-185-5p	RT primer	GTCGTGTCTGAGGCTCACTGAGACGATCGCACCCTCGACACGACTCAGGAAC
	Reverse PCR primer	CTGAGGCTCACTGAGACG
	PCR probe	(R6G)-TCGCACCC(T-BHQ1)CGACACGACTCAGGAAC
	Forward PCR primer	CTACACTGGAGAGAAAGGCA
miR-191-5p	RT primer	GTCGTGTCTGAGGCTCACTGAGACCTATTCGCACCTCGACACGACCAGCTGCT
	Reverse PCR primer	CTGAGGCTCACTGAGACCT
	PCR probe	(R6G)-ATTCGCACC(T-BHQ1)CGACACGACCAGCTGCT
	Forward PCR primer	CAGCCAACGGAATCCCAAA
miR-199b-5p	RT primer	GTCGTGTCTGAGGCTCACTGAGACCTATTCGCACCCTCGACACGACGAACAGATA
	Reverse PCR primer	CTGAGGCTCACTGAGACCT
	PCR probe	(R6G)-ATTCGCACCC(T-BHQ1)CGACACGACGAACAGATA
	Forward PCR primer	TCAGCCCCAGTGTTTAGAC
miR-26a-5p	RT primer	CGTGATGCGCCTTGTAGCACGACCTTATTCGCACCCTCGCATCACGAGCCTATC
	Reverse PCR primer	GCCTTGTAGCACGACCTTA
	PCR probe	(R6G)-TTCGCACCC(T-BHQ1)CGCATCACGAGCCTATC
	Forward PCR primer	CGAGCCATTCAAGTAATCCAG
miR-378-3p	RT primer	GTCGTGTCTGAGGCTCACTGAGACCTATTCGCACCTCGACACGACGCCTTCTG
	Reverse PCR primer	CTGAGGCTCACTGAGACCT
	PCR probe	(R6G)-ATTCGCACC(T-BHQ1)CGACACGACGCCTTCTG
	Forward PCR primer	CTGAGGCTCACTGAGACCT

miR-96-5p	RT primer	GTCGTGTCTGAGGCTCACTGAGACCTTTCGCACCCTCGACACG ACCAGCAAAAATG
	Reverse PCR primer	CTGAGGCTCACTGAGACCT
	PCR probe	(R6G)-TTCGCACCC(T-BHQ1)CGACACGACCAGCAAAAATG
	Forward PCR primer	CGGACTTTGGCACTAGCA

(+C=C-LNA; +T=T-LNA; R6G -Rhodamine 6G (Rhodamine 590); BHQ1 - Black Hole Quencher-1; LNA - Locked Nucleic Acid).

Table S3. List of microRNAs suitable for analysis.

MiRNAs suitable for analysis.	MiRNAs, the number of which were different in the comparison groups (differential expression in the comparison groups showed more than a 3-fold difference).
hsa-let-7a-5p	hsa-let-7a-5p
hsa-let-7b-5p	hsa-let-7c-5p
hsa-let-7c-5p	hsa-let-7e-5p
hsa-let-7d-5p	hsa-let-7f-5p
hsa-let-7e-5p	hsa-miR-106a-5p/miR-17-5p
hsa-let-7f-5p	hsa-miR-107
hsa-let-7g-5p	hsa-miR-1183
hsa-let-7i-5p	hsa-miR-1246
hsa-miR-106a-5p/miR-17-5p	hsa-miR-125b-5p
hsa-miR-106b-5p	hsa-miR-126-3p
hsa-miR-107	hsa-miR-1260a
hsa-miR-1183	hsa-miR-1299
hsa-miR-1246	hsa-miR-130a-3p
hsa-miR-125b-5p	hsa-miR-144-3p
hsa-miR-126-3p	hsa-miR-145-5p
hsa-miR-1260a	hsa-miR-146a-5p
hsa-miR-1299	hsa-miR-148a-3p
hsa-miR-1310a-3p	hsa-miR-148b-3p
hsa-miR-140-5p	hsa-miR-150-5p
hsa-miR-142-3p	hsa-miR-155-5p
hsa-miR-144-3p	hsa-miR-16-5p
hsa-miR-145-5p	hsa-miR-181a-5p
hsa-miR-146a-5p	hsa-miR-185-5p
hsa-miR-148a-3p	hsa-miR-18a-5p
hsa-miR-148b-3p	hsa-miR-190a-5p
hsa-miR-150-5p	hsa-miR-1910-5p
hsa-miR-155-5p	hsa-miR-194-5p
hsa-miR-15a-5p	hsa-miR-199a-3p/miR-199b-3p
hsa-miR-15b-5p	hsa-miR-199a-5p
hsa-miR-16-5p	hsa-miR-199b-5p
hsa-miR-181a-5p	hsa-miR-19a-3p
hsa-miR-185-5p	hsa-miR-20a-5p/miR-20b-5p
hsa-miR-186-5p	hsa-miR-22-3p
hsa-miR-18a-5p	hsa-miR-221-3p

hsa-miR-190a-5p<br>hsa-miR-191-5p<br>hsa-miR-1910-5p<br>hsa-miR-194-5p<br>hsa-miR-199a-3p/miR-199b-3p<br>hsa-miR-199a-5p<br>hsa-miR-199b-5p<br>hsa-miR-19a-3p<br>hsa-miR-19b-3p<br>hsa-miR-20a-5p/miR-20b-5p<br>hsa-miR-21-5p<br>hsa-miR-22-3p<br>hsa-miR-221-3p<br>hsa-miR-222-3p<br>hsa-miR-223-3p<br>hsa-miR-23a-3p<br>hsa-miR-24-3p<br>hsa-miR-25-3p<br>hsa-miR-26a-5p<br>hsa-miR-26b-5p<br>hsa-miR-29a-3p<br>hsa-miR-29b-3p<br>hsa-miR-29c-3p<br>hsa-miR-30b-5p<br>hsa-miR-30c-5p<br>hsa-miR-30d-5p<br>hsa-miR-30e-3p<br>hsa-miR-30e-5p<br>hsa-miR-32-5p<br>hsa-miR-342-3p<br>hsa-miR-361-5p<br>hsa-miR-362-5p<br>hsa-miR-363-3p<br>hsa-miR-374a-5p<br>hsa-miR-374b-5p<br>hsa-miR-376a-3p<br>hsa-miR-378<br>hsa-miR-423-5p<br>hsa-miR-424-5p<br>hsa-miR-425-5p<br>hsa-miR-4454/miR-7975<br>hsa-miR-451a<br>hsa-miR-486-3p<br>hsa-miR-491-5p<br>hsa-miR-493-3p<br>hsa-miR-506-3p<br>hsa-miR-93-5p<br>hsa-miR-96-5p<br>hsa-miR-98-5p<br>hsa-miR-99a-5p	hsa-miR-222-3p<br>hsa-miR-223-3p<br>hsa-miR-23a-3p<br>hsa-miR-24-3p<br>hsa-miR-25-3p<br>hsa-miR-26a-5p<br>hsa-miR-29a-3p<br>hsa-miR-29c-3p<br>hsa-miR-30b-5p<br>hsa-miR-30d-5p<br>hsa-miR-30e-3p<br>hsa-miR-30e-5p<br>hsa-miR-32-5p<br>hsa-miR-361-5p<br>hsa-miR-363-3p<br>hsa-miR-374b-5p<br>hsa-miR-376a-3p<br>hsa-miR-424-5p<br>hsa-miR-425-5p<br>hsa-miR-4454/miR-7975<br>hsa-miR-451a<br>hsa-miR-486-3p<br>hsa-miR-491-5p<br>hsa-miR-493-3p<br>hsa-miR-506-3p<br>hsa-miR-93-5p<br>hsa-miR-98-5p<br>hsa-miR-99a-5p

Table S4. The list of experimentally confirmed genes targeted by miRNAs. The list was generated by DIANA-TarBase v8.

miRNA	Experimentally confirmed miRNA targets
let-7a-5p	*GARS, HMGA2,DICER1, NAP1L1, CRY2, OXA1L, ZFP36, BACH1, REV3L, TRIB1, VEZT, PATL1, HMGCS1, ATP6V1F, ACTG1, PPP1R15B, NFAT5, ACTB, CNOT1, RC3H2, ZFP3CL1, SEL1L, YOD1, SKIL, COL1A2, COL3A1, TGFBR1, PAPPA, PPP1R15B, MAPK6, PXT1, CCDC71L, IRS2, ERCC6, RANBP6, PGRMC1, PDX, ARID3A, MARS2, RBFOX2, SEMA4C, SENP5, IGF2BP2, GPCPD1, SMARCC1, CERCAM, THBS1*
miR-26a-5p	*HMGA1, HMGA2, AHNAK, DDX17, TNRC6A, TNRC6B, KPNA2, MCL1, VMA21, VGLL4, YWHAE, EIF4G2, EEF2, FBXO11, TNPO1, CSK3B, LARP1, KLHL42, STYX, SLC7A11, PRKCD, NABP1, CDK8, ZDHHC6, ATAD2B, CHAC1, FRAT2, TOB1, CCDC6, FLVCR1, BAG4, MSMO1, ZSWIM6, ANKS1A, RSPRY1, TBC1D15, DDX3X, OSBPL11, PTGS2, NTN4, INHBA, HOHA9, HSPA4L, ANL2, B3GNT5, MAT2A, RLF, PIM1, TNPO1, PHHX, FAM8A1, AGPAT5, DDX3Y, SERBP1, TET1, MTPN, CREBL2, EIF4G2, ADAM10, SETD8, ATPAF1, SFPQ, DTD2, FAM46C, SFXN1, COMMD8, NCEH1, CREB1, FGF9, ANKIB1, TOP1, ZNF724P, TNKS2*
miR-181a-5p	*K MCL1, COPS2, HSPA8, ZFAND3, G3BP2, SLC38A2, SLC2A3, BUB3, CCSAP, FOS, KDELR2, RPS18, RPS4X, BHLHE40, BIRC6, CUL5, LIN28B, WASL, TBPL1, GPCPD1*
miR-185-5p	*VEGFA, EIF4EBP2, POL2R2A, PPAP2B, IGFBP5, ENAH*
miR-96-5p	*FEM1B, KPNB1, GINM1, VAMP3*
miR-199b-5p	*ELP2, MGAT4B, RASSF3*
miR-145-5p	*ACTB, FBN1, LTA4H, RAD23B*
miR-150-5p	*LDLR, PDE7A*
